# Comparative Expression Profiling of Wild Type *Drosophila* Malpighian Tubules and *von Hippel-Lindau* Haploinsufficient Mutant

**DOI:** 10.3389/fphys.2019.00619

**Published:** 2019-05-21

**Authors:** Marilena Ignesti, Davide Andrenacci, Bettina Fischer, Valeria Cavaliere, Giuseppe Gargiulo

**Affiliations:** ^1^Dipartimento di Farmacia e Biotecnologie, Alma Mater Studiorum Università di Bologna, Bologna, Italy; ^2^CNR Istituto di Genetica Molecolare, Unità di Bologna, Bologna, Italy; ^3^IRCCS, Istituto Ortopedico Rizzoli, Bologna, Italy; ^4^Department of Genetics, University of Cambridge, Cambridge, United Kingdom; ^5^Cambridge Systems Biology Centre, University of Cambridge, Cambridge, United Kingdom

**Keywords:** microarray analysis, *Drosophila VHL*, renal carcinoma, kidney, sensitized genetic background

## Introduction

The von-Hippel Lindau (VHL) disease is a hereditary genetic disorder that predisposes to the onset of several highly vascularized benign and malignant tumors, developing with elevate frequency in the central nervous system and kidneys. The most-aggressive VHL tumor is ccRCC, the clear-cell renal cell carcinoma, affecting the kidney. VHL disease etiology can be attributed to the inheritance of a *VHL* loss-of-function allele, typically a deletion (Gnarra et al., 1994; Herman et al., 1994); this facilitates the somatic inactivation of the other allele (through amorphic mutations or gene silencing through promoter methylation), leading to the onset of the tumorous phenotype (Latif et al., 1993). This reveals the haploinsufficient behavior of the *VHL* gene.

The high vascularization of VHL tumors can be explained considering that human VHL protein is the substrate-binding subunit of an E3 ubiquitin ligase (Lonergan et al., 1998; Iwai et al., 1999; Kamura et al., 1999) involved in the poly-ubiquitination of HIF-1α transcription factor. This post-translational modification leads HIF-1α to proteosomal degradation (Maxwell et al., 1999). Loss of *VHL* function causes the stabilization of HIF-1α, triggering cellular response and adaptation to hypoxic conditions (expression of genes involved in glycolysis, angiogenesis and erythropoiesis) (Bader and Hsu, 2012). While this represents the canonical function of VHL, other HIF-1α-independent function of VHL have been identified, thanks to the contribution of model organisms (Hsu, 2012). Indeed, *VHL* gene function is conserved and also *Drosophila* has a *VHL* homolog, the *dVHL* gene (Adryan et al., 2000; Aso et al., 2000). *dVHL* is involved in the development of *Drosophila* vascular system (Adryan et al., 2000; Hsouna et al., 2010) and in morphogenesis of follicular epithelium of the egg chamber (Duchi et al., 2010). Interestingly, some *VHL* functions are mediated by Awd, an endocytic mediator whose human orthologs are NME1/2 metastasis suppressors (Rosengard et al., 1989). Awd is broadly required during *Drosophila* development since it is involved in epithelial morphogenesis (Nallamothu et al., 2008; Woolworth et al., 2009; Ignesti et al., 2014) and required for maintaining genomic stability (Romani et al., 2017). Moreover, Awd is also present into the extracellular fluids of *Drosophila* larvae (Romani et al., 2016, 2018).

In *Drosophila*, two pairs of monolayered epithelial Malpighian tubules, each composed of 100-150 cells, absolve to osmoregulation and excretion functions (Denholm and Skaer, 2009). Transcriptomic analysis of Malpighian tubules revealed that among genes that are here enriched there are homologs of human genes implicated into renal pathologies (Wang et al., 2004). This justifies the use of *Drosophila* Malpighian tubules as model system to gain insights into pathophysiology of human kidneys (Dow and Romero, 2010; Miller et al., 2013).

The *dVHL*^1.1^ allele is a loss of function mutation of the *dVHL* locus (Duchi et al., 2010; Hsouna et al., 2010). *dVHL*^1.1^*/*+ flies mimic the genetic condition of VHL patients. We carried out a genome-wide gene expression profiling of whole Malpighian tubules dissected from *Drosophila* females both heterozygous for the *dVHL*^1.1^ mutation and with two wild type copies of the *dVHL* gene. The comparison of differentially expressed genes in the two genetic backgrounds potentially allows to identify genes that are sensible to *dVHL* functional copy number. Quality control assessments of the data were performed and results obtained from the differential expression analysis were confirmed by qRT-PCR. With this approach we aimed to provide a well-controlled dataset for a better understanding of the VHL disease. Indeed, even if further molecular and functional characterization are needed, human homologs of the differentially expressed genes, if existing, could have a role in the somatic inactivation of the wild type copy of *VHL* and/or into the very first phase of cancer onset.

## Materials and Methods

### *Drosophila* Stocks and Genotypes

*Drosophila* flies were raised at 25°C on a standard cornmeal/yeast/agar culture medium. We used *y*^1^*,w*^67*c*23^ flies as wild type stock. The *dVHL*^1.1^ null mutation has been previously characterized (Duchi et al., 2010; Hsouna et al., 2010).

### Malpighian Tubules RNA Extraction

Fifty *Drosophila* females of the appropriate genotype (*dVHL*^1.1^*/*+ or wild type flies) were transferred every day into vials with fresh yeasted food for 5 days. Malpighian tubules were then dissected and 400 μl of TRIzol were added. Homogenization was performed keeping samples on ice. 10 μg of linear polyacrylamide were added before centrifuging at 16,000 g (10 min). 80 μl of chloroform were added to supernatant. Sample was vortexed for 60 s and then centrifuged at 16,000 g (15 min). The upper phase was transferred to a new RNase-free tube. 0.8 volumes of isopropanol were added. RNA was then precipitated for 1 h at −20 μC and pelleted by centrifugation at 16,000 g (30 min). Pellet was then washed with 500 μl of 70% ethanol and centrifuged at 16,000g (5 min). Ethanol was then removed and pellet re-suspended in 15 μl of DEPC water. RNA concentration and purity was assessed through NanoDrop spectrophotometer.

### cDNA Generation, Amplification, and Labeling

Four biological replicates were performed. Five Hundred nana gram of RNA were amplified using the SMARTer™ PCR cDNA synthesis kit (Clontech) following manufacture's instruction. Amplified cDNA was then labeled by using the Klenow labeling of double stranded DNA protocol. The number of cycles required to obtain products in exponential phase was determined by performing a PCR using the Advantage® II PCR kit (Clontech) and following manufacture's protocol (5′ PCR Primer II used: 5′-AAGCAGTGGTATCAACGCAGAGT-3′). DNA was purified using QIAquick PCR purification columns. Nine nano gram of cDNA were labeled through incorporation of dCTP conjugated with Cy3 or Cy5 dyes using the BioPrime DNA Labeling System and following manufacture's protocol. Cy3 and Cy5 labeled sample and control pairs were combined in 1.5 ml tubes. The volume was reduced to 25–30 μl in a SpeedVac concentrator before proceeding with Sephadex G50 purification (two per sample), assembled following manufacture's instruction. Sample volumes was reduced to 2–5 μl using a SpeedVac. Finally, 2 μl of 10 mg/ml sonicated salmon sperm DNA were added with 140 μl of hybridization buffer. Samples were then boiled at 100°C (2 min), centrifuged at 16,000 × g (1 min) and then hybridized on slides.

### FL003 Array Hybridization

The FlyChip in-house printed FL003 gene expression arrays on FMB PowerMatrix slides using the Genetix Qarray2 (producing 82 arrays per run), consisting of 14,444 transcript-specific 70-mer oligonucleotides were used (GEO accession GPL14121). Four biological replicates were performed including 2 dye swaps. Blocking of slides was performed (as per FMB protocol) by incubating slides for 30 min in 0.1% BSA, 0.2% SDS, 2x SSC (300 mM NaCl, 30 mM Na citrate, pH 7), followed by three washes in clean water. One hundred and thirty five microliters of samples were hybridized for 16 h at 51°C with agitation using the GeneTac Hybridisation station. Slides were then washed with pre-warmed (55°C) wash solution 1 (0.2 × SSC; 0.2% SDS) for 20 min with gentle agitation, followed by 3 washes for 1 min in warm solution 2 (0.2 × SSC), avoiding light exposure, rinsed with MilliQ water at room temperature and finally dried in a centrifuge at 96 × g (5 min).

### Data Acquisition and Processing

Slides were scanned using an Axon GenePix 4000B scanner at optimal PMT gain. Manual spot-finding was operated through Dapple (Buhler et al., 2000). Raw data was imported into limma (Bioconductor R package, R version 3.1.0) and Variance stabilizing normalization (vsn) was applied (Huber et al., 2002). Significance analysis was performed using the empirical Bayes method within limma. Due to the low number of significant genes (*n* = 8 at fdr <= 0.05) thresholds were relaxed to include genes with average M value <-0.5 or >0.5 (M-value is the log2 of the ratio of sample vs. control intensities), and *p*-value <0.1 (187 genes).

### Quantitative RT-PCR Analysis

Three biological replicates of Malpighian tubules dissected from 30 females were analyzed. Malpighian tubule total RNA was extracted in TRI Reagent (Sigma-Aldrich) and treated with TURBO DNase (Ambion). RNA was reverse transcribed using the high-capacity RNA-to-cDNA kit (Applied Biosystems) according to the manufacture's protocol. Quantitative real-time PCRs were performed in fast 48-well reaction plates (Applied Biosystems) and analyzed by StepOnePlus real-time PCR system (Applied Biosystems) according to the manufacturer's procedure. For each sample, at least two technical replicates were performed. Primers were designed using Primer 3 software (Untergasser et al., 2012). Parameters for primer design were a length of 18-27 bases, a melting temperature between 57.0-63.0°C, and a GC content from 20-80%. *dGrip75* and *CG31955* primers were designed in different exons. Expression of target genes was normalized to the widely used reference gene *Rp49*. The qRT-PCR primers used are listed in Table S1. For each gene of interest, fold changes in expression levels were evaluated by using the ΔΔCt method. The mean fold change and SD were calculated. *p*-value was calculated using a one-tail *t*-test analysis on three biological replicates. Dissociation curve analysis was performed to confirm the presence of a single specific product.

## Differentially Expressed Genes in *dVHL^1.1^/+* Malpighian Tubules

**Figure 1:**
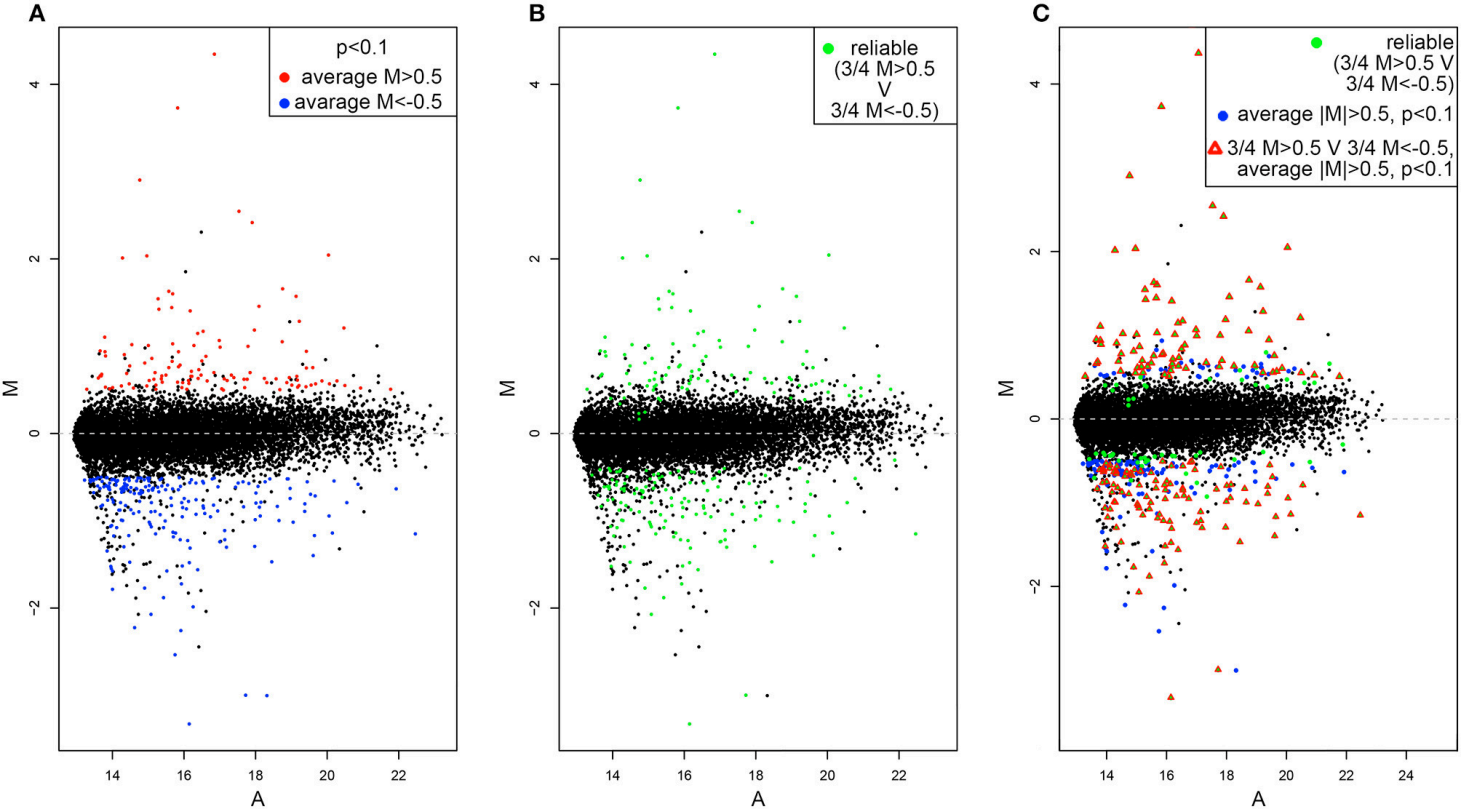
MA-plots of differentially expressed genes in *dVHL*^1.1^*/*+ Malpighian tubules. **(A)** Upregulated genes (average M > 0.5, *p* < 0.1) are shown in red while downregulated genes (average M<-0.5, *p* < 0.1) are shown in blue. **(B)** Genes for which the alteration in gene expression (M > 0.5 or M<-0.5) was recovered in 3 out of 4 slides are shown in green. **(C)** Genes that satisfy both the cut-off conditions in **(A,B)** are shown with red triangle.

By using the statistical parameters reported in the material and methods section we recovered 331 hit genes whose expression significantly differ between *dVHL*^1.1^*/*+ and wild type tubules (Figure 1A). One hundred and eighteen are upregulated (red dots) while 321 are downregulated (blue dots). The majority of genes are not significantly differentially expressed (black dots), as expected. By looking at M values of genes in each of the 4 slides we also highlighted those of them for which single absolute M values are higher than 0.5 in at least 3 slides (Figure 1B). This should outline genes for which the alteration in gene expression is reliable (based on alteration reproducibility). Finally, we merged the data in Figures 1A,B and found 187 genes for which the absolute value of average M is higher than 0.5, *p*-value is lower than 0.1 and in at least 3 out of 4 slides the single absolute M-values are higher than 0.5 (Figure 1C).

**Figure 2:**
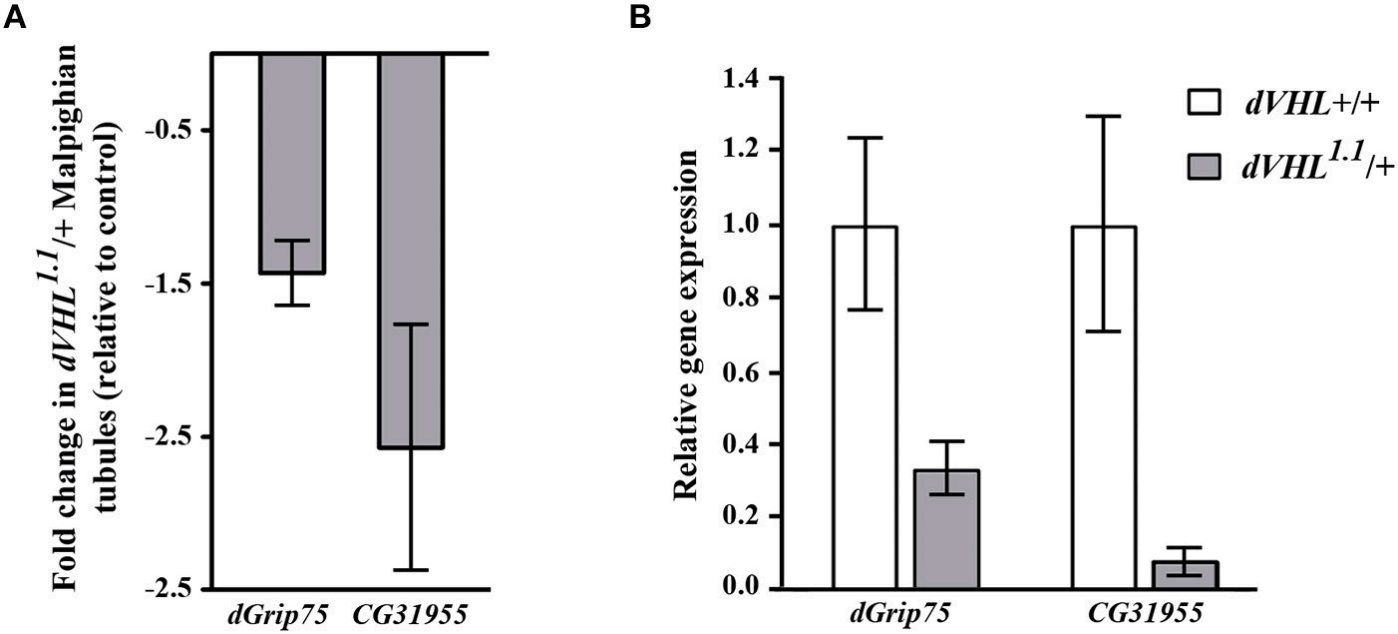
Validation and classification of differentially expressed genes in *dVHL*^1.1^*/*+ Malpighian tubules. **(A)** Fold change in transcription levels of *dGrip75* and *CG31955* genes in the microarray analysis. **(B)** qRT-PCR analysis of *dGrip75* and *CG31955* transcript levels in individuals of the reported genotypes. 3 biological replicates of tubules from 5-days old *dVHL*^1.1^/+ and wild type females were analyzed. Graphs represent mean ± SD; *p* = 0.0152 (*dGrip75)* and *p* = 0.0153 *(CG31955)*.

As an initial step to analyze the differentially expressed genes we performed quantitative real time PCR (qRT-PCR) experiments and we analyzed the transcript levels of two genes that we are interested on studying, *dGrip75* and *CG31955*; *rp49* was used as internal reference gene (Figure 2B). The qRT-PCR experiments confirmed that, in *dVHL*^1.1^*/*+ Malpighian tubules, both genes are downregulated, as expected by microarray results (Figure 2A). *dGrip75* encodes a γ-tubulin which takes part in the assembly of the γ-tubulin ring complex (γTuRC), located at the centrosomes, at the base of a microtubule. γTuRC has a ring-shaped structure that serves as a template for a microtubule and allows the controlled polymerization of tubulin dimers (Oegema et al., 1999; Moritz et al., 2000). This protein attracted our attention since we have already demonstrated that dVHL is essential in follicle cells via stabilizing microtubules (Duchi et al., 2010). *CG31955* encodes a protein with unknown molecular function. An interesting microarray study of Andrew (Chung et al., 2011) showed that *CG31955* is downregulated in *trachealess (trh)* mutant embryos. Trh is the master regulator of trachea development, the *Drosophila* branched and tubular system which is responsible for transport of oxygen and other gases. Earlier analysis on *dVHL* highlighted its requirement in this tubular organ: heterozygous and homozygous *dVHL*^1.1^ embryos display altered tracheal system (Hsouna et al., 2010).

We screened our candidate list with FlyMine (Lyne et al., 2007) tool for gene ontology (GO) enrichment in biological processes (*p* < 0.1, Bonferroni test) and we found enrichment in regulation of phosphoprotein phosphatase activity [GO:0043666, *p*-value = 0.05] and regulation of protein serine/threonine phosphatase activity [GO:0080163, *p* = 0.07].

Transcriptome alterations in human morphologically normal cells heterozygous for a *VHL* mutation (derived from VHL patients) have also been analyzed (Peri et al., 2016). A comparison between *Drosophila* and human datasets could recover strong hits, whose molecular dissection may be performed using *Drosophila* as a model system.

The limiting-most aspect of this study is intrinsic to omics approaches: functional analyses of candidates are needed to genetically dissect gene functions and pathways, confirming their role into the VHL pathogenesis.

## Data Availability

The datasets generated for this study can be found in the GEO data repository (http://www.ncbi.nlm.nih.gov/geo/) under the accession identification number GSE124152.

## Author Contributions

MI cultured flies and performed the experiments. BF assisted the microarray experiments. DA performed the qPCR analysis. MI, GG, and VC conceived the experiments and wrote the article.

### Conflict of Interest Statement

The authors declare that the research was conducted in the absence of any commercial or financial relationships that could be construed as a potential conflict of interest.
