# Supplementary material for: Comparative Expression Profiling of Wild Type Drosophila Malpighian Tubules and von Hippel-Lindau Haploinsufficient Mutant
Source: Front Physiol. 2019 May 21;10:619. doi: 10.3389/fphys.2019.00619 (PMC6547062; doi:10.3389/fphys.2019.00619)
Supplement: Supplementary file 1 [file Table_1.DOCX]

| Gene  name | Primers sequences (5’-3’) | Experimental Tm (°C) | Y-intercept | R^2^ | Efficiency (%) |
| --- | --- | --- | --- | --- | --- |
| *dGrip75* | fw TTGGAGTTCTGCTCCCAAAT  re TCATCTGTGATTCCCGTGAC | 78.0 | 21,0 | 0,9977 | 93,74 |
| *CG31955* | fw GTGAGCCCAGAAAACAGAGC  re GCGTTCTCACGTGTTCCTTT | 79.3 | 22,7 | 0,9967 | 98,71 |
| *Rp49* | fw TCTGCATGAGCAGGACCTC  re ATCGGTTACGGATCGAACAA | 83.3 | 21,9 | 0,9990 | 95,29 |

Gene name, primer sequence, experimental Tm of the amplicon, Y-intercept, R^2^, and efficiency of the primers. The calibration curve was obtained by diluting a 10 ng of DNA stock with a serial factor of 1:5.

Table S1. List of primers used in this study.
